# Supplementary material for: L-Methionine Protects against Oxidative Stress and Mitochondrial Dysfunction in an In Vitro Model of Parkinson’s Disease
Source: Antioxidants (Basel). 2021 Sep 15;10(9):1467. doi: 10.3390/antiox10091467 (PMC8469212; doi:10.3390/antiox10091467)
Supplement: Supplementary file 1 [file antioxidants-10-01467-s001.zip › antioxidants-1340554-supplementary.pdf]

Supplementary Materials

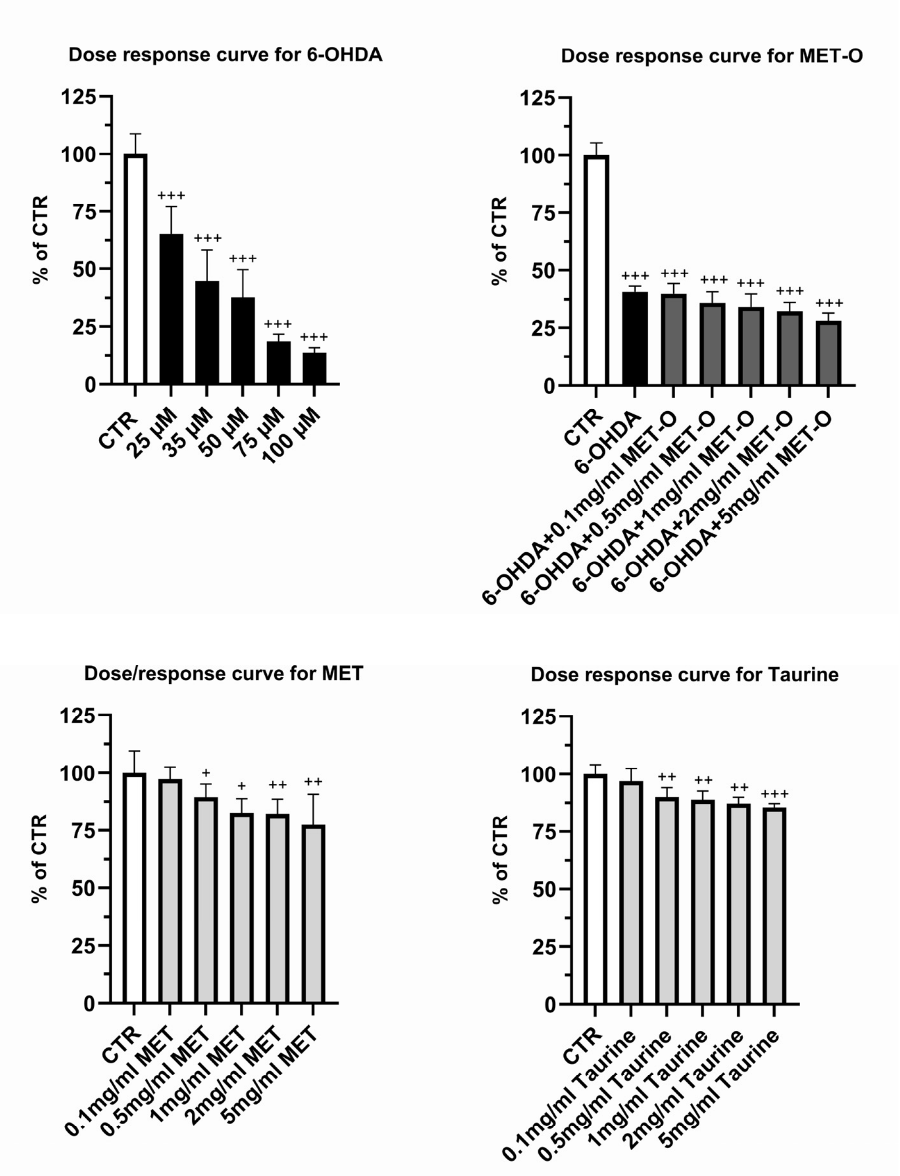

Figure S1. Dose response curve for Met, Met-O, taurine and 6-OHDA. Data are mean ± SD of 3 different experiments. Data are expressed as % of CTR. +++ $p < 0.0001$; ++ $p < 0.005$; + $p < 0.05$ vs. CTR.

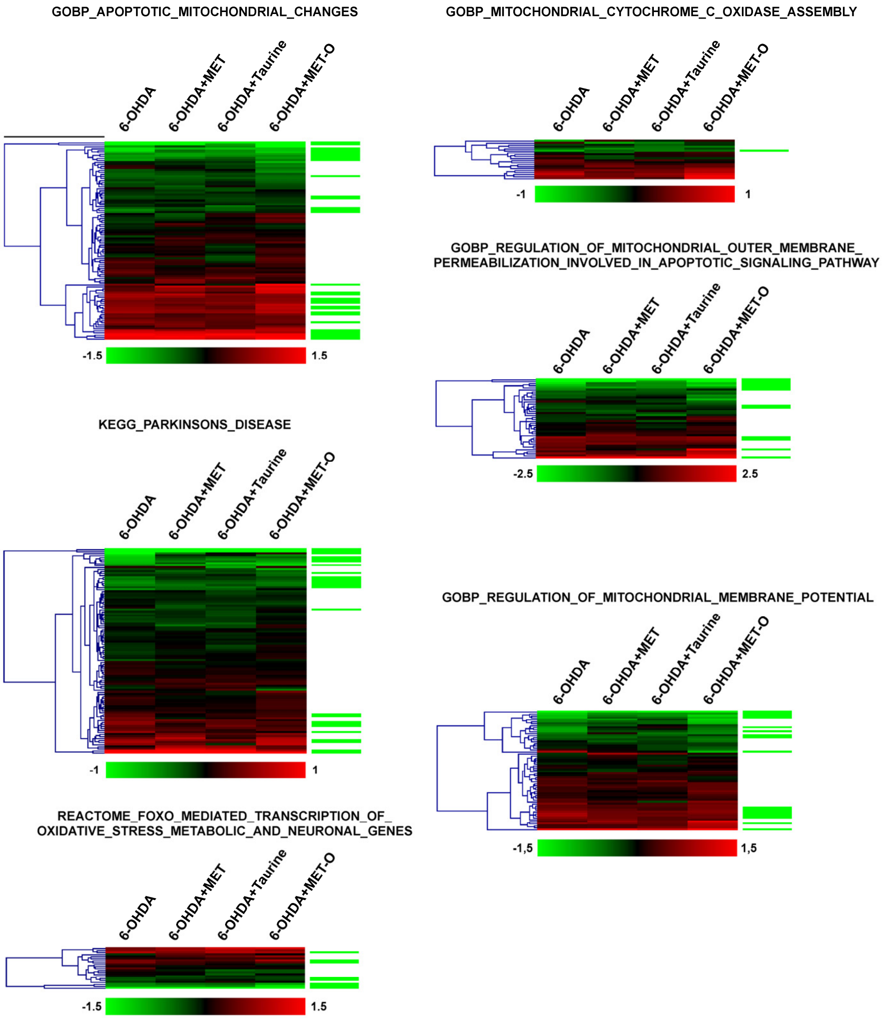

Figure S2. Heatmap of hierarchical clustering of the selected pathways. Color scale represents log2 ratios of the expression levels in the indicated condition versus control. Color scale limits are indicated in the boxes below the respective heatmap.

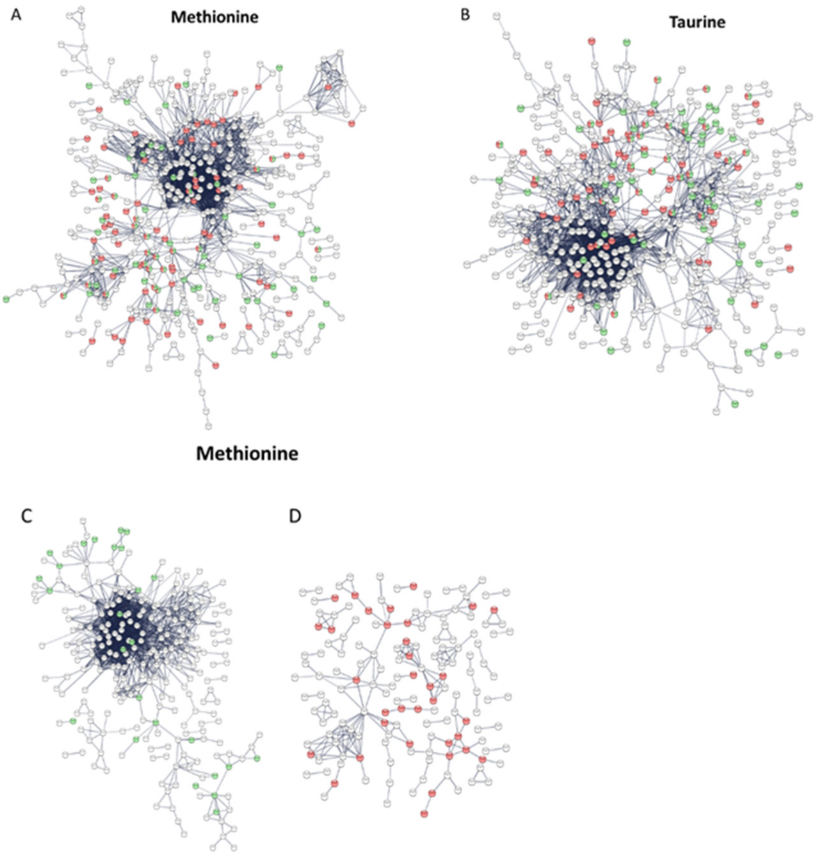

Figure S3. String protein network analysis. Upper panels. The analysis reveals that Methionine (**A**) and taurine (**B**) can modulate the expression of targets involved in apoptosis and cell death processes (red) and neurogenesis (green). Lower panels. Protein interactions associated with neurogenesis (green) and apoptosis/cell death (red) are mainly among the genes respectively down- (**C**) or up- (**D**) regulated by 6-OHDA and recovered by Methionine.

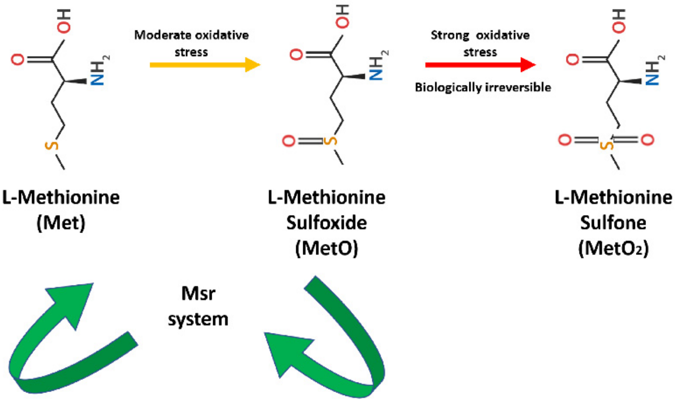

Figure S4. Schematic figure representative of the Mrs system which reduce methionine sulfoxide to methionine repairing its antioxidant activity.

Table S1. Significance data relative to TMRM analyses (**Figure 9**) at different time points.

**vs CTR**					
**0**					
CTR vs. 6-OHDA	0	-0,1794 to 0,1794	No	ns	>0,9999
CTR vs. 6-OHDA+MET	0	-0,1794 to 0,1794	No	ns	>0,9999
CTR vs. 6-OHDA+MET-O	0	-0,1794 to 0,1794	No	ns	>0,9999
CTR vs. 6-OHDA+TAU	0	-0,1794 to 0,1794	No	ns	>0,9999
**4**					
CTR vs. 6-OHDA	0,6259	0,4465 to 0,8052	Yes	****	<0,0001
CTR vs. 6-OHDA+MET	0,2125	0,03315 to 0,3919	Yes	*	0,0151
CTR vs. 6-OHDA+MET-O	0,4413	0,2619 to 0,6207	Yes	****	<0,0001
CTR vs. 6-OHDA+TAU	0,1374	-0,04197 to 0,3168	No	ns	0,1799
**8**					
CTR vs. 6-OHDA	0,7037	0,5244 to 0,8831	Yes	****	<0,0001
CTR vs. 6-OHDA+MET	0,2207	0,04131 to 0,4001	Yes	*	0,0111
CTR vs. 6-OHDA+MET-O	0,5826	0,4033 to 0,7620	Yes	****	<0,0001
CTR vs. 6-OHDA+TAU	0,2514	0,07200 to 0,4308	Yes	**	0,0031
**12**					
CTR vs. 6-OHDA	0,8434	0,6640 to 1,023	Yes	****	<0,0001
CTR vs. 6-OHDA+MET	0,2387	0,05936 to 0,4181	Yes	**	0,0053
CTR vs. 6-OHDA+MET-O	0,787	0,6076 to 0,9664	Yes	****	<0,0001
CTR vs. 6-OHDA+TAU	0,2731	0,09374 to 0,4525	Yes	**	0,0012
**16**					
CTR vs. 6-OHDA	0,932	0,7527 to 1,111	Yes	****	<0,0001
CTR vs. 6-OHDA+MET	0,3723	0,1930 to 0,5517	Yes	****	<0,0001
CTR vs. 6-OHDA+MET-O	0,8869	0,7075 to 1,066	Yes	****	<0,0001
CTR vs. 6-OHDA+TAU	0,463	0,2836 to 0,6424	Yes	****	<0,0001
**24**					
CTR vs. 6-OHDA	1,006	0,8262 to 1,185	Yes	****	<0,0001
CTR vs. 6-OHDA+MET	0,434	0,2546 to 0,6134	Yes	****	<0,0001
CTR vs. 6-OHDA+MET-O	0,9713	0,7919 to 1,151	Yes	****	<0,0001
CTR vs. 6-OHDA+TAU	0,5068	0,3274 to 0,6862	Yes	****	<0,0001

**vs 6-OHDA**					
**0**					
6-OHDA vs. CTR	0	-0,1794 to 0,1794	No	ns	>0,9999
6-OHDA vs. 6-OHDA+MET	0	-0,1794 to 0,1794	No	ns	>0,9999
6-OHDA vs. 6-OHDA+MET-O	0	-0,1794 to 0,1794	No	ns	>0,9999
6-OHDA vs. 6-OHDA+TAU	0	-0,1794 to 0,1794	No	ns	>0,9999
**4**					
6-OHDA vs. CTR	-0,6259	-0,8052 to -0,4465	Yes	****	<0,0001
6-OHDA vs. 6-OHDA+MET	-0,4133	-0,5927 to -0,2340	Yes	****	<0,0001
6-OHDA vs. 6-OHDA+MET-O	-0,1845	-0,3639 to -0,005153	Yes	*	0,042
6-OHDA vs. 6-OHDA+TAU	-0,4885	-0,6678 to -0,3091	Yes	****	<0,0001
**8**					
6-OHDA vs. CTR	-0,7037	-0,8831 to -0,5244	Yes	****	<0,0001
6-OHDA vs. 6-OHDA+MET	-0,483	-0,6624 to -0,3037	Yes	****	<0,0001
6-OHDA vs. 6-OHDA+MET-O	-0,1211	-0,3005 to 0,05829	No	ns	0,2738
6-OHDA vs. 6-OHDA+TAU	-0,4524	-0,6317 to -0,2730	Yes	****	<0,0001
**12**					
6-OHDA vs. CTR	-0,8434	-1,023 to -0,6640	Yes	****	<0,0001
6-OHDA vs. 6-OHDA+MET	-0,6047	-0,7841 to -0,4253	Yes	****	<0,0001
6-OHDA vs. 6-OHDA+MET-O	-0,05644	-0,2358 to 0,1229	No	ns	0,8458
6-OHDA vs. 6-OHDA+TAU	-0,5703	-0,7497 to -0,3909	Yes	****	<0,0001
**16**					
6-OHDA vs. CTR	-0,932	-1,111 to -0,7527	Yes	****	<0,0001
6-OHDA vs. 6-OHDA+MET	-0,5597	-0,7391 to -0,3803	Yes	****	<0,0001
6-OHDA vs. 6-OHDA+MET-O	-0,04518	-0,2246 to 0,1342	No	ns	0,9217
6-OHDA vs. 6-OHDA+TAU	-0,469	-0,6484 to -0,2897	Yes	****	<0,0001
**24**					
6-OHDA vs. CTR	-1,006	-1,185 to -0,8262	Yes	****	<0,0001
6-OHDA vs. 6-OHDA+MET	-0,5716	-0,7509 to -0,3922	Yes	****	<0,0001
6-OHDA vs. 6-OHDA+MET-O	-0,03424	-0,2136 to 0,1451	No	ns	0,9694
6-OHDA vs. 6-OHDA+TAU	-0,4988	-0,6781 to -0,3194	Yes	****	<0,0001

TAU=taurine.
